# Facilitating cancer systems epidemiology research

**DOI:** 10.1371/journal.pone.0255328

**Published:** 2021-12-31

**Authors:** Rolando Barajas, Brionna Hair, Gabriel Lai, Melissa Rotunno, Marissa M. Shams-White, Elizabeth M. Gillanders, Leah E. Mechanic

**Affiliations:** 1 Epidemiology and Genomics Research Program, Division of Cancer Control and Population Sciences (DCCPS), National Cancer Institute (NCI), National Institutes of Health (NIH), Bethesda, Maryland, United States of America; 2 DCCPS, NCI, NIH, Bethesda, Maryland, United States of America; Georgia State University, UNITED STATES

## Abstract

Systems epidemiology offers a more comprehensive and holistic approach to studies of cancer in populations by considering high dimensionality measures from multiple domains, assessing the inter-relationships among risk factors, and considering changes over time. These approaches offer a framework to account for the complexity of cancer and contribute to a broader understanding of the disease. Therefore, NCI sponsored a workshop in February 2019 to facilitate discussion about the opportunities and challenges of the application of systems epidemiology approaches for cancer research. Eight key themes emerged from the discussion: transdisciplinary collaboration and a problem-based approach; methods and modeling considerations; interpretation, validation, and evaluation of models; data needs and opportunities; sharing of data and models; enhanced training practices; dissemination of systems models; and building a systems epidemiology community. This manuscript summarizes these themes, highlights opportunities for cancer systems epidemiology research, outlines ways to foster this research area, and introduces a collection of papers, “Cancer System Epidemiology Insights and Future Opportunities” that highlight findings based on systems epidemiology approaches.

## Introduction

Epidemiology research has been successful at identifying many risk factors for complex diseases such as cancer, but much of the etiology remains unexplained. This may be due, in part, to the limited focus of many studies on a small number of risk factors or contributors to disease within specific domains or measures. Moreover, many studies fail to evaluate the complexities and interrelations among multiple risk factors on each other and the study outcomes. Each individual risk factor, such as a single dietary component or genetic polymorphism, occurs in a broader biological or societal context that may modulate the effect of individual risk factors on cancer. Many risk factors for cancer are also highly correlated with possible interactive, additive, synergistic, or attenuating effects. Additionally, many risk factors are dynamic and time-varying: changes over the life course and the timing of exposure may modify cancer risk [1, 2].

Several groups have advocated for a more holistic or comprehensive analytic approach to the study of disease in populations [3–10]. This type of approach may lead to a better understanding of the mechanisms of disease and has been described by investigators using terminologies such as: eco-epidemiology [11], populomics [12], globolomics [13], systems medicine [14], and systems epidemiology [13, 15–18]. The term “systems epidemiology” conceptually borrows from fields such as systems biology, considering epidemiology research in a systems framework. For the purposes of this paper, we define systems epidemiology as an approach to study risk and outcomes that incorporates high-dimensional measurements from multiple domains, assesses the inter-relationships between risk factors, and considers changes over time. Our definition was adapted from Damman (2014) [17], however, we emphasize the importance of dynamism within a systems approach. Systems epidemiology research may leverage advanced computational simulation and modeling techniques to assess these complex networks and perform comprehensive analyses. Importantly, systems approaches are not limited to any single analytical method, but constitute a framework to account for complexity and understand the broader context of disease [7, 17, 19, 20].

Due to the complexity of cancer, a systems epidemiology approach may complement more traditional methods and lead to insights in disease etiology. To facilitate discussion about the application of systems modeling approaches for cancer epidemiology, NCI sponsored a workshop in February 2019 (https://epi.grants.cancer.gov/events/systems-epidemiology/) with presentations and discussions by experts in diverse fields to gain broader perspectives (S1 Table). In this manuscript, we summarize eight major themes from the workshop that will facilitate systems epidemiology research (Table 1) and discuss opportunities for this approach as exemplified by the accompanying papers in this PLOS Collection, “Cancer System Epidemiology Insights and Future Opportunities”.

**Table 1:** Major themes identified to advance systems epidemiology research.

Theme	Description
**1. Transdisciplinary Collaboration and a Problem-Based Approach**	The ability to perform systems epidemiology research is contingent on the engagement of experts from varying fields to holistically address a scientific problem. Needs for transdisciplinary collaboration included: encouraging a focus on research problems holistically, bringing researchers together, addressing communication barriers, and sustaining transdisciplinary collaboration.
**2. Methods and Modeling Considerations**	Whether data-driven or hypothesis-driven, the overall methodology for systems epidemiology must incorporate an iterative approach where models evolve over time based on results. Several methods exist to apply systems modeling. Newer improved methods should incorporate changes over time, bridging multiple scales (e.g., cell, individual, and neighborhood), and dealing with unknown contributions of chance.
**3. Interpretation, Validation, and Evaluation of Models**	The complexity of systems models results in challenges for interpretation, validation, and evaluation. Comparative modeling, using common datasets or controls, and reproducibility pipelines are possible strategies to address these issues.
**4. Data Needs and Opportunities**	Despite numerous rich datasets in support of epidemiology research, data gaps remain. These gaps include the need for data from populations underrepresented in biomedical sciences, health behaviors, built environment, and health care provider information. Opportunities exist to leverage data from wearable devices, electronic health records, and large cohorts and initiatives. Challenges were noted regarding combining data from multiple sources and research domains.
**5. Sharing of Data and Models**	Promotion of systems epidemiology depends on the ability to share models and data. Effective sharing and reuse requires sufficient documentation and mechanisms to assess quality and support findability. Some mechanisms and infrastructures, including existing sharing platforms, could be leveraged to help address these needs.
**6. Enhanced Training Practices**	The evolving field of systems epidemiology will need to facilitate training for both students and current researchers in systems modeling, transdisciplinary research, data sciences, informatics, and computational modeling.
**7. Dissemination of Systems Models**	Successful dissemination depends on effective communication with content experts and the non-research community. Through direct engagement of various stakeholders, systems methods are more likely to be translated, utilized, and accepted to inform biological interpretations, interventions, or policies.
**8. Building a Systems Community**	Sustainability of systems epidemiology may depend on cultivating a systems epidemiology community. This can be facilitated by establishing organizations, interest groups, or other platforms for sharing ideas and discussing models. Specialized funding initiatives and review panels may further support systems epidemiology research.

## Themes to facilitate systems epidemiology research

### 1. Transdisciplinary collaboration and a problem-based approach

To more holistically study cancer, collaboration across disciplines is required. Traditionally, there has been a tendency when studying complex diseases for researchers to focus on data from individual disciplines. Focusing on a problem-based approach could bridge scientists across disciplines and integrate unique perspectives to improve understanding [21, 22]. Specifically, systems epidemiology would benefit from building linkages between disease content experts and computational modelers and informaticists who can build informed computational models.

There are several examples of how transdisciplinary collaborations and a problem-based approach can lead to scientific insights. In one transdisciplinary collaboration, researchers developed improved methods of differentiating between benign and aggressive cancer lesions [23]. Transdisciplinary collaboration also allows for methods to be developed and shared across fields. For example, dynamic agent-based modeling was developed for infectious disease modeling but now is commonly used in other complex disease analyses in public health [2, 24].

Several mechanisms, including previous NIH funding initiatives [21, 25–30], have encouraged transdisciplinary collaborations and problem-based approaches, which may serve as models to support this type of work. Another opportunity to foster transdisciplinary collaborations is to bring researchers together prior to applying for research funding. This type of process was used by the Cancer Research UK-NCI “Sandpit” workshop [31] and National Science Foundation ideas labs [32].

Challenges to be considered in developing transdisciplinary collaborations include sustainability and lack of a shared language among different scientific fields. Individuals with familiarity or training in multiple disciplines can serve as “translators” or “connectors” and facilitate interactions between distinct fields.

### 2. Methods and modeling considerations

Two complementary strategies were discussed to support systems epidemiology research: hypothesis-driven and data-driven strategies. In a hypothesis-driven strategy, researchers focus on the data necessary to address a specified scientific hypothesis and analyze the data to test that hypothesis. In a data-driven strategy, the most likely hypothesis is identified based on a more agnostic, algorithm-based data exploration of several hypotheses. If the goal is to understand the overall mechanism, a hypothesis-driven strategy may be preferable. At the same time, given that a hypothesis-driven strategy is limited by current knowledge and assumptions, a data-driven strategy may gain new knowledge by finding unexpected relationships through a more agnostic approach.

The application of a systems approach to epidemiology questions should be considered an iterative process involving several steps, including identifying the problem, determining the model to test, obtaining the data, analyzing results, refining the model, and repeating as necessary based on the results. At times, these steps can occur concurrently. An example of how modeling was used to inform data collection was demonstrated by the Cancer Intervention and Surveillance Modeling Network (CISNET) breast consortium. Using simulation modeling, the CISNET teams examined the need for radiotherapy in women assessed as low risk based on genomic testing. These results were useful for informing the design of clinical trials by identifying those populations where data from the trial would be most informative [33].

Applying a systems approach to epidemiology research may be supported by several types of existing methods including systems dynamics, network analysis, agent-based modeling, and others [19, 24, 34–41] (S2 Table). Regardless of the specific analytical method, the unique aspect of a systems epidemiology approach is accounting for the complexity of the system by considering multiple domains, inter-relationships between risk factors, and dynamism. Needs for additional methods were noted, in particular for models incorporating time and space, dealing with the unknown contribution of chance, and bridging multiple scales (e.g., protein, cell, tissue, individual, neighborhood, community, ecosystem). It is critical for researchers to understand the underlying assumptions in methods and the strengths or weaknesses of particular models for different situations and questions. The variety of methods makes it challenging to interpret and compare results obtained using different approaches. Therefore, an important component of applying systems approaches is the validation and evaluation of models.

### 3. Interpretation, validation, and evaluation of models

As the complexity of a model increases with additional variables, the sparsity of data increases, thereby reducing the ability to make predictions or classifications. The large number of attributes in a model can also result in overfitting, which leads to biases in a model and makes it difficult to generalize or apply the model to another population. These issues with complex modeling are often referred to as the curse of dimensionality problem [42]. Furthermore, deep epidemiology data, including repeated measurements over time and assessments of multiple domains, is usually only available on smaller populations, limiting generalizability to other populations. Therefore, to adequately interpret the data and assess causality using these models, an iterative process is needed that includes well-designed validation and evaluation steps which will lead to model refinement and attenuation of these issues.

Model validation is the process of checking if all technical aspects (e.g., parameters definitions, coding, etc.) are done adequately or need refining and is preferably performed by an independent party [43]. Model evaluation is the process of assessing the performance and reproducibility of a complete model to discover its likelihood to perform in real world conditions (e.g., training and testing, cross-validation, etc.) [44]. To implement the validation and evaluation of their models, CISNET uses a comparative modeling approach where multiple research groups examine the same research questions using different models and identical predictors, and evaluate the results against real data trends. The consistency across models provides support for model predictions [45]. When comparing results from complex models, care must be taken in the selection of the evaluation metric or the control used for comparison purposes. The dataset selected as a control could be biased in favor of the model being evaluated based on assumptions inherent in the model and the control dataset. Evaluating these complex models using common control datasets [46] could reduce this potential issue.

Given the complexity of methods for systems approaches, it may be challenging to reproduce all aspects of the analysis. One possible solution suggested at the workshop was to develop a reproducibility pipeline, or clear documentation for other researchers to apply the models on other populations. Notably, lack of reproducibility may also be due to intrinsic differences in the studied populations (e.g., by racial/ethnic or exposures distributions) [46]. Fortunately, there is guidance for validating and evaluating complex models [43, 47]. Further emphasis on the best practices for application of systems models to epidemiology research may help advance the use of these models in epidemiology studies.

### 4. Data needs and opportunities

Sufficient data (real and simulated) is required to effectively characterize a system [15]. Though workshop participants identified several potential data resources which could be used to support systems epidemiology research (S3 Table), gaps remain. One critical gap is the lack of inclusion of understudied groups, including racial and ethnic, socioeconomic, and geographic diversity and sexual/gender minorities [48, 49]. Insufficient racial and ethnic diversity is also apparent in genomics research [50, 51] and genomic catalogues [52, 53].

Other needs and opportunities identified by participants were for quality information about health behaviors, the built environment, and health care provider data. Systems epidemiology research could be enhanced by improving access or utilization of data sources such as: wearable devices (i.e. Fitbits), electronic health records [54], and large initiatives such as the All of Us cohort [55] and Environmental Influences on Child Health Outcomes (ECHO) Program [56]. As the understanding of a system develops and new hypotheses emerge, data needs may change. Collecting broad and multiple data types may enable the examination of multiple hypotheses without going back to data collection, which is particularly challenging in population-based studies. Such a strategy was used by the Community of Mine study [57]. Moreover, biobanks linked to medical record data provide another potential resource for systems epidemiology research [58] and could be leveraged to estimate risk factor or biomarker distributions in a target population missing that information [59, 60].

Characterizing the system requires combining or integrating several sources of data such as measures from different domains (e.g. genetics and behavioral) or spatial (e.g. cell to tissue) and temporal (e.g. day vs. year) scales. Often data is formatted uniquely, stored with different levels of metadata, or located in diverse databases. In fact, it was suggested that the resources required for integrating diverse, large-scale data types surpasses the resources required for generating these data [61]. For multi-omic data, several software frameworks have been developed to address some of these challenges, including Galaxy, Taverna, KNIME, and bioKepler [62]. Additional work is needed in this area.

### 5. Sharing of data and models

Improved methods for data linkages and model sharing across disciplines can facilitate systems epidemiology research by enabling a) analyses incorporating information from multiple domains; b) validation and evaluation of models and results; c) efficiency by avoiding duplication of efforts. Effective sharing and reuse of data and models requires adequate documentation (including metadata and descriptors) and mechanisms to assess quality and findability [63], which can be costly. The NIH has worked to provide additional funding support for data and model sharing [64–67]. Moreover, according to the NIH Genomic Data Sharing Policy (GDS), costs for sharing of data should be included in the project budget [68]. Implementing carefully curated datasets or model resources and standardizing data quality indicators can increase confidence in methods and aid reproducibility and reusability. To address difficulty in finding the appropriate data set or method (i.e. findability), databases or resources that list and describe models are needed, such as the NCI Genetic Simulation Resources [69]. Several platforms and infrastructures were discussed that support sharing data and/or analytical models (Table 2).

**Table 2:** Example platforms supporting sharing of data and analytical models or methods.

Data Sharing Platforms/Solutions	Description
All of Us Research Workbench	The All of Us cohort has a goal of enrolling one million participants in the United States to improve health sciences research. With the goal of oversampling for diverse participants, the data being collected includes various measures such as: health questionnaires, electronic health records (EHRs), physical measurements, the use of digital health technology, and biospecimens.
Link: https://www.researchallofus.org/workbench/	
Database of Genotypes and Phenotypes (dbGaP)	dbGaP stores and distributes results of studies that have investigated the association between genotypes and phenotypes.
Link: https://www.ncbi.nlm.nih.gov/gap/	
NCI Cancer Research Data Commons (CRDC)	The NCI Cancer Research Data Commons (CRDC) is a cloud-based data science infrastructure that connects data sets with analytics tools to allow users to share, integrate, analyze, and visualize cancer research data.
Link: https://datascience.cancer.gov/data-commons	
Model Sharing platforms/Analysis platforms	Description
Bioconductor	Bioconductor provides tools for the analysis and comprehension of high-throughput genomic data.
Link: https://www.bioconductor.org/	
Galaxy	Galaxy is a web-based platform that enables multi-omics data integration and analysis workflows.
Link: https://usegalaxy.org/	
Kepler project (and bioKepler)	Kepler is designed to harmonize data by allowing scientists to create, execute, and share models and analyses across a broad range of scientific disciplines.
Link: https://kepler-project.org/	
KNIME	KNIME is an analytics platform that supports data science workflows and reusable components.
Link: https://www.knime.com/	
Taverna	Taverna is a suite of tools used to design and execute scientific workflows.
Link: https://taverna.incubator.apache.org/introduction/	
Combined Analysis and Data platforms	Description
Biosphere	Biosphere is an open-source platform developed by the Broad Institute that can operate across several different platforms (e.g., Terra, Gen3, and Dockstore) to create an interoperable data environment for the biomedical community.
Link: https://www.databiosphere.org/	
Genomic Data Science Analysis, Visualization, and Informatics Lab-space (AnVIL)	AnVIL is a scalable and interoperable resource for the genomic scientific community. It leverages a cloud-based infrastructure for genomic data access, sharing and computing across various data sets.
Link: https://www.genome.gov/Funded-Programs-Projects/Computational-Genomics-and-Data-Science-Program/Genomic-Analysis-Visualization-Informatics-Lab-space-AnVIL#overview	
NCI Cloud Resources	The NCI Cloud Resources are components of the NCI Cancer Research Data Commons that allow researchers to download, store, and analyze vast datasets in the cloud. The platform gives users access to tools and pipelines already implemented or lets them upload their own data or analytical methods to workspaces.
Link: https://datascience.cancer.gov/data-commons/cloud-resources	
St. Jude Cloud-based Repository	St. Jude Cloud is a large pediatric genomics dataset that offers a suite of unique analysis tools and visualizations. It supports access to more than 700 paired tumor/germline samples for common and rare pediatric cancers, sequenced as part of the Pediatric Cancer Genome Project (PCGP).
Link: https://www.stjude.cloud/	
Terra	Terra is a scalable and secure platform for biomedical researchers to access Broad hosted data, upload their own data, and combine data to run on analytic tools. The platform also contains functions that promote sharing of data to facilitate collaboration between scientists.
Link: https://terra.bio/	

### 6. Enhanced training practices

Participants noted that encouraging a systems framework for epidemiology research will require improvements in training, including more opportunities focused on systems science such as the *Systems Science for Social Impact* program [70]. Meeting participants suggested changes to the current epidemiology academic curriculum to incorporate systems training with more emphasis on complexity, transdisciplinary research, computational modeling, and informatics throughout the training continuum for epidemiologists. The current academic infrastructure is designed to develop scientists that are experts in specific fields/disciplines [22, 71], whereas systems approaches to epidemiology research require breadth of training across disciplines. Several training programs have supported this multidisciplinary model [72–74]. Another important training need is in the areas of data science, informatics, and computational modeling, particularly for population scientists. Training the next generation of data scientists and integrating these researchers into biomedical and public health fields is a priority within the NIH strategic plan for data science [75]. Finally, continuing education programs for epidemiologists [76], along the lines of Continuing Medical Education (CME) course work for physicians, could also broaden use of systems methods. Topics suggested for this type of training included advanced modeling techniques and managing and interpreting uncertainty in models.

### 7. Dissemination of systems models

For systems epidemiology modeling to be useful for research and policy, models and results using these methods need to be disseminated and accepted.

Stakeholders (e.g., patients, providers, payers, policy makers) should be involved early in model development to inform parameters and priorities. Incorporating stakeholder feedback can improve model quality by better defining the system and increase stakeholders’ adoption of such models. Obtaining feedback on models as they are being developed through early publication may also lead to better models. However, journals may be reluctant to publish conceptual models in the absence of application results. A venue allowing for publication of early conceptual models could promote feedback (e.g., the preprint server bioRxiv https://www.biorxiv.org/).

Another key component to dissemination of models is effectively communicating models to the community for researchers, clinicians, policymakers, and the general public. Making the results interpretable regardless of model complexity would build confidence in the model and results [77]. Moreover, it is important to explain that uncertainty in the results remains even though systems models are sophisticated [78]. Effective communication could be enhanced by encouraging media training for scientists.

### 8. Building a systems epidemiology community

Growth in the application of systems approaches to epidemiology research will require building a community of systems epidemiology researchers. Workshop participants noted that the current workshop was unique and expressed enthusiasm for bringing together researchers from disparate fields to address complex problems. In addition to periodic in-person meetings or workshops such as the one described by this paper, one strategy to build this type of community is to establish organizations, interest groups, or social platforms that can bring many different scientists together to share ideas and discuss and compare models, such as the Interdisciplinary Association for Public Health Science (IAPHS) [79]. Opportunities to promote cross-fertilization of ideas would be a systems epidemiology-focused journal, or a journal collection such as this one, where researchers from different disciplines can publish papers in this arena.

Another strategy to build a systems epidemiology community is through tailored grant reviews and specialized funding opportunities. Some meeting participants suggested that the non-linear design and the multidisciplinarity underlying complex modeling and systems approaches do not easily fit into the traditional three-aim structure of R01 applications, making it more challenging for this type of research to compete for funding. Special funding opportunities that support more complex projects and are amenable to non-linear aims, feedback loops and iterative processes may be helpful for this field. Special review panels for systems epidemiology applications could include reviewers from different disciplines, with at least one reviewer with computational modeling expertise, assigned to review each application.

## Opportunities for systems epidemiology research

In addition to the eight themes highlighted which would facilitate systems epidemiology, several research opportunities that may be addressed using a systems epidemiology approach were discussed by workshop participants (Table 3).

**Table 3:** Example opportunities for a systems epidemiology approach in cancer research.

Major Opportunity Areas	Example Research Questions
**Understanding the complexity of common risk factors:** Risk factors that have remained elusive in their contribution to cancer etiology can be studied systematically.	• Study obesity via a systems approach to discover the dynamic (feedback/feedforward) role obesity (both child and adult) has on cancer etiology and survivorship. • The systemic effects of circadian rhythm disruptions on behaviors, organ physiology, and metabolism that could better explain cancer etiology. • Estimate the uncertainty of exposure measurements (i.e., contributed from measurement errors, high background rates for some contaminants, challenges in assessing mixtures) in causal relationships and better understand contribution to disease.
**Integration of environmental/behavioral factors:** Study the interaction between individual health behaviors and environments that increase cancer risk.	• Behaviors are often assessed individually and in absence of environmental context. However, a systems approach can support the evaluation of how factors like sexual behaviors, nutrition, tobacco usage, physical activity, sedentary behavior, circadian rhythm (sleep) disruptions, social networks, and infectious disease transmission work in tandem and vary within different environments (e.g., rural vs. urban settings) to contribute to cancer.
**Health inequalities/health disparities:** Evaluate how health inequalities are reflected throughout various biological, socioeconomic, and environmental layers.	• Certain groups may have a higher risk of certain cancers due to many factors such as stress, low access to care, education, environmental exposures, and genetics. • Disentangle the independent and interrelated contributions of genetic ancestry and socially defined race/ethnicity to cancer.
**Improvement of cancer therapies:** Incorporate social determinants of health and other high-dimensional population-based measurements from multiple domains (e.g., neighborhood pollution and physical activity) to inform differences in treatment response.	• Examine how dynamic social determinants (e.g., diet, lifestyle, environmental exposures, behaviors, etc.) work in conjunction with biological processes (e.g., the microbiome) to influence treatment responses.
**Effective interventions and screening (i.e., design, predict, and evaluate interventions):** Integrate knowledge about biology and the embedded context of individuals to provide personalized intervention strategies and screening programs.	• Utilize a systems approach to better prioritize at-risk populations and tailor interventions beyond a single behavior. • Evaluate and further improve interventions and screening programs over time in the specific environment/population of interest.
**Policy Impacts:** Examine the government/policy/institutional systemic impacts on cancer risk, and how policies may be improved via a systems approach.	• Use system modeling approaches to test a policy prior to implementation or examine impact of policy under different conditions. • Examine the impact of a policy change on an outcome, accounting for unforeseen consequences and feedback loops. • Evaluate the long-term impacts of a policy, accounting for changes in components of a system (e.g., changes in behaviors or movement of populations).

One research opportunity that received substantial attention was to use systems approaches to help understand and alleviate health disparities. Complex social, behavioral, environmental, biological, and ecological contributions to disparities vary by context, impact multiple scales, and involve nonlinear and multidirectional associations (or feedback loops). The systems nature of health disparities may explain their persistence across different diseases. A systems approach may thus provide valuable insights into the etiology of disparities to highlight sources of inequities, identify data needs, and improve interventions [4].

These research opportunities and the papers in this “Cancer System Epidemiology Insights and Future Opportunities” collection illustrate the promise of systems epidemiology approaches. However, a portfolio analysis by Shams-White et al. found that despite specific systems and computational modeling funding announcements, the representation of systems epidemiology grants in cancer research remains low [80]. Together the above examples and these results suggest that many cancer-related research questions addressable using a systems approach may therefore benefit from tailored funding opportunities.

## Conclusions and next steps

At the outset of the workshop, several participants expressed uncertainty about the definition of systems epidemiology. Nevertheless, there was overall agreement about the need for the general approach. Some participants suggested that it was important to emphasize the time element, or dynamism, within the definition as changes over time are critical to consider and are often missing in traditional studies. Others underlined the importance of data as the availability of high throughput data can help support more systems-based approaches.

To conclude, workshop participants supported a more comprehensive approach to population-based research studies and identified several considerations to facilitate the field of systems epidemiology. The workshop identified several themes or considerations for facilitating systems epidemiology research and exemplified research opportunities. These themes included: transdisciplinary collaboration and a problem-based approach; methods and modeling considerations; interpretation, validation, and evaluation of models; data needs and opportunities; sharing of data and models; enhanced training practices; dissemination of systems models; and building a systems epidemiology community. As a first step to continue the conversation, several researchers participated in this collection of papers, outlining research opportunities and findings using systems epidemiology approaches. Our intent is that this collection will further spark discussion and foster continued research in this area.

## Supporting information

S1 Table Expertise of workshop participants. (DOCX)

S2 Table Example methods applicable for systems epidemiology. (DOCX)

S3 Table Example data resources applicable for systems epidemiology. (DOCX)
