# Supplementary material for: Facilitating cancer systems epidemiology research
Source: PLoS One. 2021 Dec 31;16(12):e0255328. doi: 10.1371/journal.pone.0255328 (PMC8719747; doi:10.1371/journal.pone.0255328)
Supplement: S1 Table — (DOCX) [file pone.0255328.s001.docx]

Supplemental Table 1: Expertise of Workshop Participants

| Areas of Expertise | Number of participants that reported this expertise |
| --- | --- |
| Bioinformatics and Computational Biology | 6 |
| Cancer Survivorship | 4 |
| Cell Biology | 0 |
| Clinical and Medical Science | 3 |
| Computational Modeling | 4 |
| Engineering | 2 |
| Epidemiology | 11 |
| Genetics/Genomics | 4 |
| Genomic Epidemiology | 3 |
| Health Disparities | 10 |
| Molecular Biology | 0 |
| Pharmacoepidemiology and Pharmacogenomics | 1 |
| Public Health Policy | 4 |
| Social Epidemiology | 4 |
| Statistics/Biostatistics | 5 |
| Systems Biology | 3 |
| Translational Medicine | 3 |
| Other: Behavioral Medicine | 1 |
| Other: (General) Systems Theory | 1 |

^a^ Workshop participants received a survey in advance of the meeting. The “Areas of expertise” were programmed options in the survey except for “Other” which was an open text field. Participants could select multiple areas of expertise.
