# Supplementary material for: Facilitating cancer systems epidemiology research
Source: PLoS One. 2021 Dec 31;16(12):e0255328. doi: 10.1371/journal.pone.0255328 (PMC8719747; doi:10.1371/journal.pone.0255328)
Supplement: S2 Table — (DOCX) [file pone.0255328.s002.docx]

Supplemental Table 2: Example Methods Applicable for Systems Epidemiology

| **Category of Method** | **Example Method** |
| --- | --- |
| Mapping | Category Theory |
|  | Concept mapping |
|  | Dialogue (qualitative) and mapping |
| Simulations | Agent based modeling |
|  | Group model building using system dynamics |
|  | Simulation models, modeling & simulation |
|  | System dynamics/systems dynamics modeling |
|  | Discrete event simulation models |
|  | Microsimulation |
| Networks | Network analysis |
|  | Neural networks |
|  | Bayesian network analysis |
|  | Social network analysis |
| Hierarchical | Bayesian hierarchical modeling |
|  | Hierarchical Models |
|  | Regularized regression |
| Mediation | Causal Inference |
|  | Mediation |
|  | Mendelian randomization |
| Clustering | Latent Variable Analysis or Models |
|  | LUCIDus: Latent Unknown Clustering with Integrated Data |
|  | Unsupervised clustering |
| Broad methods | Mathematical modeling |
|  | Machine learning |
|  | Analyzing multilevel data |
|  | Heuristic, large-scale computational experiments |
|  | Integrative genetic analyses |
| Standard approaches | Generalized linear modeling |
|  | Genetic admixture |
|  | Multivariate analysis (spec. of SEER data) |
| Data linkage and harmonization | Data linkages |
|  | Harmonizing clinical data from multiple health systems |
| Broad disciplines | Transdisciplinary/Team Science approaches |
|  | Bioinformatics |
|  | Environmental epidemiology |
|  | Genetic epidemiology |
|  | Imaging |
|  | In situ proteomics |
