# Supplementary material for: Facilitating cancer systems epidemiology research
Source: PLoS One. 2021 Dec 31;16(12):e0255328. doi: 10.1371/journal.pone.0255328 (PMC8719747; doi:10.1371/journal.pone.0255328)
Supplement: S3 Table — (DOCX) [file pone.0255328.s003.docx]

Supplemental Table 3: Example Data Resources Applicable for Systems Epidemiology

| Database | Description | Links |
| --- | --- | --- |
| A Framework for Reconstructing Epidemiological Dynamics (FRED) | An agent-based modeling system to create synthetic populations from Public use microdata and aggregated census data. | https://fred.publichealth.pitt.edu/ |
| All of Us Cohort Study | A comprehensive 1-million-person cohort based in the United States. The cohort has a special emphasis in diverse populations and collects a number of demographic, epidemiological, clinical, and biological measures. | https://allofus.nih.gov/ |
| American Time Use Survey | A survey supported by the U.S. Bureau of Labor Statistics that measures the amount of time participants spend doing various work or social activities. | https://www.bls.gov/tus/home.htm |
| Cancer Intervention and Surveillance Modeling Network (CISNET) | A consortium of National Cancer Institute (NCI)-sponsored investigators and projects that use complex simulation modeling toward research in various cancers. Modeling methods and data are shared across the consortium. | https://cisnet.cancer.gov/ |
| Catalogue of Surveillance Systems | A list of available surveillance datasets supported by the National Collaborative on Childhood Obesity Research (NCCOR). | https://tools.nccor.org/css/ |
| Centers for Medicare & Medicaid (CMS) Data | Data from the U.S that contains information on national health care programs, Medicare populations, health care utilization, expenditures, and providers. | https://data.cms.gov/ |
| Clinical Proteomic Tumor Analysis Consortium (CPTAC) | An NCI supported consortium with the aim of improving cancer etiology research through proteomic and genomic analysis. Data (genomics, proteomics, imaging, and assays) are available to the public. | https://proteomics.cancer.gov/programs/cptac |
| Database of genotypes and phenotypes (dbGaP) | An archive database that hosts data from studies that study the interaction between genotypes and phenotypes across the disease spectrum. Controlled access data can range from epidemiological to biological. | https://www.ncbi.nlm.nih.gov/gap/ |
| Genotype-Tissue Expression (GTEx) | A resource created by the Broad Institute that aims to study tissue-specific gene expression and regulation through 54 non-disease tissues from 1k participants. Data includes molecular assays and histology images. | https://www.gtexportal.org/home/ |
| Human Microbiome Project | A National Institutes of Health (NIH) Common Fund project that collects and analyzes data on the interaction between the human microbiome and various diseases. | https://commonfund.nih.gov/hmp |
| Metabolomics Workbench | A NIH Common Fund project that acts as a repository of national and international metabolomics data. It also provides analytical tools and access to metabolite study protocols/methods. | https://www.metabolomicsworkbench.org/ |
| National Health Interview Survey | A nationally representative survey of households that is collected annually. Data collected are used in various epidemiology studies and to inform public health policy. | https://www.cdc.gov/nchs/nhis/index.htm |
| NCI Cohort Consortium | A collection of cohorts supported by the NCI to foster collaborations and pool data and biospecimens to create more impactful cancer studies. | https://epi.grants.cancer.gov/cohort-consortium/ |
| New York City-Public Metropolitan Transportation Authority (MTA) Data | Subway, bus, rail, bridge, and tunnel data from the Metropolitan Transportation Authority of the State of New York. | https://data.cityofnewyork.us/Transportation/MTA-Data/mmu8-8w8b |
| RTI Synthetic Household Population | A synthetic population creation tool that provides accurate sociodemographic representation of the complete household and person population of the United States. Data is collected and integrated from public sources. | https://www.rti.org/impact/rti-us-synthetic-household-population%E2%84%A2 |
| Surveillance, Epidemiology, and End Results (SEER) | An NCI-sponsored surveillance system that monitors the cancer burden (national incidence and mortality) of the U.S. population. | https://seer.cancer.gov/ |
| Synthetic Patient Generation | A MITRE corporation funded open source tool to generate an accurate synthetic patient population to be used in health care research. The synthetic population and individual patients are modeled to encompass various diseases/conditions. | https://synthetichealth.github.io/synthea/ |
| Synthetic Populations and Ecosystems for the World (SPEW) | A R package for generating synthetic populations and ecosystems for any geographic regions in the globe. | http://www.stat.cmu.edu/~spew/img/use_spew.html |
| The Cancer Genome Atlas (TCGA) | A seminal cancer genomics research project that contains extensive genomic, clinical, and demographic information culminating into 2.5 petabytes of data. | https://www.cancer.gov/about-nci/organization/ccg/research/structural-genomics/tcga |
| The Connect Study | A prospective cohort of 200,000 adults in the U.S. designed. Leveraging new technology to measure behaviors and the environment along with biological and clinical measures, to evaluate the cancer etiology and outcomes. | https://dceg.cancer.gov/research/who-we-study/cohorts/connect |
| The Sisters study | Supported by the National Institute of Environmental Health Sciences (NIEHS), this cohort contains over 50,000 women in the US that had a sister with breast cancer. Information collected includes various socio-economic, demographic, and biological measures. | https://sisterstudy.niehs.nih.gov/English/index1.htm |
| UK Biobank | A massive national (United Kingdom) database with over 500,000 people enrolled. Epidemiological data is collected and linked to a myriad of disease with various biological measures also collected. | https://www.ukbiobank.ac.uk/ |
| United States Census Data | Demographic data from the U.S. census that represents the entire U.S. population. | https://www.census.gov/ |
| Utah population database | A state-wide, family-rich database. It contains a variety of demographic, genetic, and clinical data for millions of people. | https://uofuhealth.utah.edu/huntsman/utah-population-database/ |
